# No evidence for intervention-associated DNA methylation changes in monocytes of patients with posttraumatic stress disorder

**DOI:** 10.1038/s41598-022-22177-1

**Published:** 2022-10-17

**Authors:** Elisabeth Hummel, Magdeldin Elgizouli, Maurizio Sicorello, Elsa Leitão, Jasmin Beygo, Christopher Schröder, Michael Zeschnigk, Svenja Müller, Stephan Herpertz, Dirk Moser, Henrik Kessler, Bernhard Horsthemke, Robert Kumsta

**Affiliations:** 1 Department of Genetic Psychology, Faculty of Psychology, Ruhr University Bochum, Bochum, Germany; 2 Institute of Human Genetics, University of Duisburg-Essen, University Hospital Essen, Essen, Germany; 3 Department of Psychosomatic Medicine and Psychotherapy, Medical Faculty Mannheim, Central Institute of Mental Health, Heidelberg University, Mannheim, Germany; 4 Genome Informatics, Institute of Human Genetics, University of Duisburg-Essen, University Hospital Essen, Essen, Germany; 5 Department of Psychosomatic Medicine and Psychotherapy, LWL-University Hospital, Ruhr University Bochum, Bochum, Germany; 6 Department of Behavioural and Cognitive Sciences, Laboratory for Stress and Gene-Environment Interplay, University of Luxembourg—Campus Belval, Maison Des Sciences Humaines, 11, Porte Des Sciences, L-4366 Esch-Sur-Alzette, Luxemburg

**Keywords:** DNA methylation, Prognostic markers, Psychology

## Abstract

DNA methylation patterns can be responsive to environmental influences. This observation has sparked interest in the potential for psychological interventions to influence epigenetic processes. Recent studies have observed correlations between DNA methylation changes and therapy outcome. However, most did not control for changes in cell composition. This study had two aims: first, we sought to replicate therapy-associated changes in DNA methylation of commonly assessed candidate genes in isolated monocytes from 60 female patients with post-traumatic stress disorder (PTSD). Our second, exploratory goal was to identify novel genomic regions with substantial pre-to-post intervention DNA methylation changes by performing whole-genome bisulfite sequencing (WGBS) in two patients with PTSD. Equivalence testing and Bayesian analyses provided evidence against physiologically meaningful intervention-associated DNA methylation changes in monocytes of PTSD patients in commonly investigated target genes (*NR3C1, FKBP5, SLC6A4, OXTR)*. Furthermore, WGBS yielded only a limited set of candidate regions with suggestive evidence of differential DNA methylation pre- to post-therapy. These differential DNA methylation patterns did not prove replicable when investigated in the entire cohort. We conclude that there is no evidence for major, recurrent intervention-associated DNA methylation changes in the investigated genes in monocytes of patients with PTSD.

## Introduction

The concept of an environmentally regulated epigenome has attracted considerable attention in the mental health field^1^. Many psychopathologies have developmental origins and epigenetic processes have been suggested as targets for the effects of psychosocial adversity particularly in early life. The epigenome is an umbrella term for a range of mechanisms involved in gene regulation, including DNA methylation, histone modifications and regulation through non-coding RNA molecules^2^. Epigenetic processes are essential for normal cellular differentiation and development. Perturbations in these processes have been linked to different pathologies^3^, including mental disorders^4^. Both animal models and human studies have pointed to persistent epigenetic changes in response to various stimuli. These changes, it has been argued, might reflect or mediate the long-term effects of early risk exposure such as prenatal malnutrition on metabolic outcomes^5^. Another often quoted example of epigenetic mediation is the programming of the neuroendocrine stress response by the degree of maternal care in rodents^6^.

DNA methylation, which involves methylation of cytosines predominantly in cytosine-guanine (CpG) dinucleotides, has long been thought to represent an early-established and rather stable epigenetic marker^7–9^. A number of recent studies have shown, however, that DNA methylation patterns are more dynamic than previously thought and can change in response to internal signals or environmental influences^10–14^. These observations have sparked interest in the potential for psychological interventions to influence these biological processes. Several studies have been published that assessed DNA methylation before and after therapeutic intervention (see^15^ for review). Most followed a candidate-gene approach and investigated genes involved in stress regulation, such as the *Glucocorticoid Receptor* (*NR3C1*) or *FKBP Prolyl Isomerase 5* (*FKBP5*)^16–18^, or genes commonly investigated in psychiatric genetics studies, such as the *Serotonin Transporter* (*SLC6A4*^19^), *Monoamine Oxidase A* (*MAOA*^20^), and the *Brain Derived Neurotrophic Factor* (*BDNF*^21^). The studies conducted so far are very heterogeneous in terms of investigated disorders (post-traumatic stress disorder, PTSD; anxiety disorders, borderline personality disorder), analyzed tissue (buccal cells, whole blood, peripheral blood mononuclear cells (PBMCs), as well as type of intervention and methodology for exploring DNA methylation. A common feature of all investigations is the observation that therapy responders and non-responders showed differential DNA methylation patterns pre- to post-intervention. For instance, in combat veterans with PTSD, DNA methylation levels of *FKBP5* in PBMC decreased in responders but increased in non-responders comparing pre- to post-therapy measurements^18^. The first epigenome-wide investigation of intervention-associated DNA methylation changes in PTSD further supported this notion. Successful psychotherapeutic treatment of PTSD was associated with increases in DNA methylation of the *Zinc Finger Protein 57* gene (*ZFP57*) in whole blood, whereas DNA methylation in this region decreased during the development of PTSD, and also decreased in patients who did not respond to therapy^22^.

Taken together, DNA methylation changes have thus been suggested as a marker or epigenetic correlate of therapy outcome. Caution is needed in the interpretation of these findings though. With exception of the epigenome-wide association study, only a limited number of CpGs were investigated, the observed changes were small, and the applied methods mostly had insufficient sensitivity to reliably assess such small DNA methylation differences. Furthermore, it is unclear whether differences in DNA methylation, as observed after the intervention, do not rather reflect changes in the cellular composition of the investigated tissue. DNA methylation patterns are highly cell type-specific, and each cell type within a tissue contributes to DNA methylation variation^23^. Given that the cell composition of the circulating leukocyte pool is dynamic and influenced by various external factors, including infections, menstrual cycle^24^ or stress exposure^25^, DNA methylation changes in blood collected at different time points from the same individual might primarily reflect differences in cell composition^26^, unrelated to the effect of intervention.

To address these confounding factors, we set out to investigate intervention-associated changes in DNA methylation in a homogenous cell population (CD14^+^ monocytes), in a female-only PTSD patient cohort. Monocytes were chosen as previous studies have shown that among the heterogeneous leukocyte population, monocytes were the most sensitive subtype for traumatic experiences and variation of psychosocial conditions^27^. In addition to altered gene expression of inflammatory genes and genes involved in neurodegeneration, altered DNA methylation was also found in monocytes in patients with stress related disorders^28–30^. Monocytes are thought to functions as an important interface between the brain and the immune system, as they respond to stressors by releasing pro-inflammatory cytokines into the bloodstream and can migrate to the brain and trigger acute inflammatory processes^31–33^.

The two goals of this study were, first, to investigate whether findings from previous reported intervention-associated candidate genes (*NR3C1, FKBP5, SLC6A4, Oxytocin Receptor* (*OXTR*) could be replicated reducing confounding effects in a female-only PTSD cohort, and, second to identify new differentially methylated regions (DMRs) by performing an exploratory whole-genome bisulfite sequencing (WGBS) in a sub-sample of the PTSD cohort. DNA methylation of commonly assessed candidate genes and promising targets from WGBS were analyzed using targeted deep bisulfite sequencing (DBS). Our approach thus addressed essential confounders, namely sex, cell heterogeneity, genetic variation, coverage of the genome and coverage of CpGs within candidate genes.

## Results

### Study 1—Targeted analysis of previously investigated candidate genes

To address our first goal of replicating intervention-associated DNA methylation changes of previously assessed candidate genes, we investigated sixty female PTSD patients. Patients with PTSD had a clinically relevant reduction in PTSD symptoms, with a mean reduction of 15.6 ± 14.6 (SD) on the PTSD-Check List for DSM-5 (PCL-5)^34^. Thirty-six patients were classified as responders according to PCL-5 (drop by 10 points from pre- to post-treatment). The responders showed a mean difference between post- and pre-treatment in PCL-5 of − 24.1 ± 10.3, while the non-responders (n = 21, 35%) showed a mean difference of − 1.0 ± 7.8 (PCL-5 data was missing for three patients).

**Figure 1:**
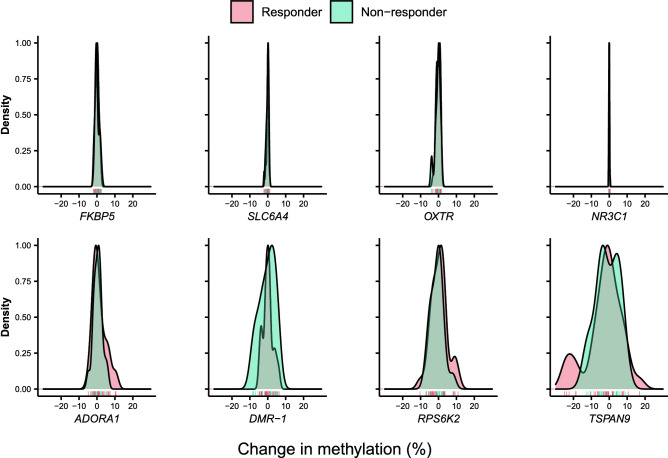
Density plots for the distribution of DNA methylation change (post minus pre-treatment) by therapy response and gene. The top row pictures the candidate genes and the bottom row the new targets from the WGBS analysis. The Y-axis shows the density and the X-axis the mean DNA methylation change in percent (%). The red curve displays the responders and the green curve the non-responders. The individual patients are depicted as red and green lines on the X-axis.

**Figure 2:**
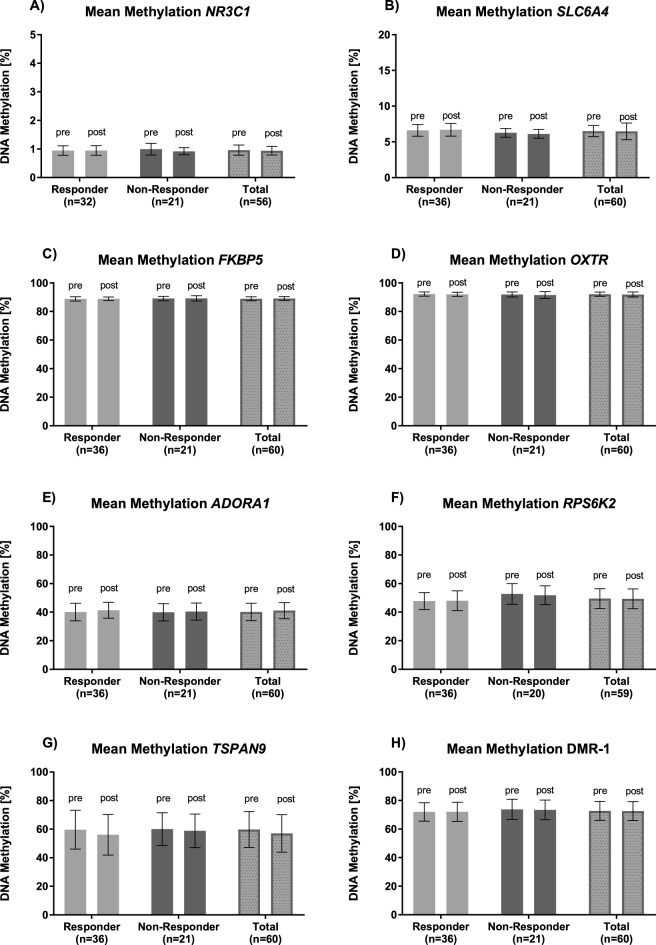
Mean DNA Methylation of the PTSD cohort. Mean DNA methylation values in percent (%) of candidate genes (**A–D**) and new targets from WGBS (**E–H**) pre-and post-intervention for responders, non-responders and the entire cohort are shown.

DBS was performed for four candidate genes for which psychotherapy effects on DNA methylation change have been previously reported: *NR3C1*, *FKBP5*, *SLC6A4* and *OXTR* (see Supplementary Fig. S1 for exact chromosomal locations). Mean DNA methylation for each gene was determined and further analyzed for treatment-associated changes. Linear regression analysis showed no significant association between pre-treatment mean DNA methylation of DBS targets and the severity of baseline PTSD symptoms (Supplementary Table S1). Likewise, there were no statistically significant differences in mean DNA methylation between pre- and post-intervention for all investigated targets, even without correction for multiple comparisons (all p > 0.05; Table 1). Bayes factors favored the null hypothesis of no mean DNA methylation change for *NR3C1* and *FKBP5.* Furthermore, mean DNA methylation change was not conditional on therapy response (all p > 0.16; Table 1), with all Bayes factors larger than one and Bayes factors for *SLC6A4* and *FKBP5* above the threshold of three. Moreover, we observed no statistically significant correlation between symptom changes and mean DNA methylation changes (all p > 0.15; Table 2 and Supplementary Fig. S2, upper row), with all Bayes factors larger than one and Bayes factors for *SLC6A4* and *FKBP5* above three. The distribution of DNA methylation changes is shown in Fig. 1, upper row by responder status. The largest mean difference between time points was found for *OXTR* (− 0.26 ± 1.21%), and the smallest was − 0.019% ± 0.19% for *NR3C1*. Mean DNA methylation levels pre- and post-intervention averaged across the analyzed genomic regions are shown in Fig. 2A–D.

**Table 1:** Effect of *intervention* and the effect of *intervention* by *responder* status interaction on DNA methylation.

	Intervention				Intervention x responder						
	*F*	*p*	$\eta_{{\text{G}}}^{2}$	*BF*_01_	*F*		*p*		$\eta_{{\text{G}}}^{2}$		*BF*_01_
**Candidate genes**											
*NR3C1*	1.72	0.196	0.01	**3.05**	1.94		0.170		0.01		1.58
*SLC6A4*	1.25	0.268	0.00	2.69	0.01		0.926		0.00		**3.65**
*OXTR*	2.77	0.102	0.01	1.85	0.58		0.449		0.00		2.78
*FKBP5*	0.47	0.494	0.00	**4.12**	0.08		0.784		0.00		**3.69**
**New targets from WGBS**											
*ADORA1*	3.52	0.065	0.01	0.64	0.61		0.439		0.00		2.75
*TSPAN9*	3.22	0.078	0.01	0.74	0.84		0.363		0.00		2.43
*RPS6KA2*	0.15	0.701	0.00	**5.19**	1.06		0.308		0.00		2.63
DMR-1	0.07	0.790	0.00	**5.06**	0.22		0.640		0.00		**3.34**

Results from a mixed model analysis of variance. ƞ^2^_G_ = generalized eta squared. *BF*_01_ = Bayes factor quantifying the evidence for the null hypothesis against the alternative hypothesis. Bayes factors above 3 are interpreted as substantial evidence for the null hypothesis and shown in boldface. Numerator *df* = 1 for all tests. Denominator *df* for *NR3C1* = 51 and for *RPS6KA2* = 54 due to missing values. For all other genes denominator *df* = 55.

**Table 2:** Correlations between change in DNA methylation and change in symptom scores.

Gene	*r*	95% CI	*p*	*BF*_01_
**Candidate genes**				
*NR3C1*	− 0.17	[− 0.42, 0.11]	0.235	1.70
*SLC6A4*	− 0.02	[− 0.28, 0.24]	0.874	**3.30**
*OXTR*	0.11	[− 0.16, 0.36]	0.425	2.50
*FKBP5*	− 0.04	[− 0.30, 0.22]	0.778	**3.22**
**New targets from WGBS**				
*ADORA1*	− 0.19	[− 0.43, 0.07]	0.155	1.32
*TSPAN9*	0.16	[− 0.11, 0.40]	0.242	1.78
*RPS6KA2*	− 0.12	[− 0.37, 0.14]	0.365	2.28
DMR-1	− 0.10	[− 0.35, 0.17]	0.473	2.64

*r* = Pearson's correlation coefficient. _0_ For *NR3C1*, *n* = 53. For *RPS6KA2*, *n* = 56. For all remaining genes, *n* = 57. *BF*_01_ = Bayes factor quantifying the evidence for the null hypothesis against the alternative hypothesis. Bayes factor above 3 is interpreted as substantial evidence for the null hypothesis and shown in boldface.

Complementary to the Bayes factor approach, equivalence testing assisted in inferring whether true population effect sizes are smaller than a pre-defined smallest effect size of interest. Effect sizes for all candidate genes were significantly smaller than 1% DNA methylation change, even in the therapy-responder group and after Bonferroni correction (all p_corrected_ < 0.001). The 95% confidence intervals of difference scores in the responder group did not include DNA methylation changes larger than ≈|0.5%|: Δ_FKBP5_ = 0.06% [− 0.28, 0.40], Δ_SLC6A4_ = − 0.11% [− 0.33, 0.11], Δ_OXTR_ = − 0.16% [− 0.52, 0.21], Δ_NR3C1_ = 0.00% [− 0.06, 0.07]. We conclude that true effect sizes of all classic candidate genes are smaller than 1% DNA methylation change.

Furthermore, taking into account common genetic polymorphisms in *FKBP5* (rs1360780) and *SLC6A4* (promoter linked polymorphic region, 5-HTTLPR), there were no associations between genotype and DNA methylation or genotype and symptom severity at pre-treatment. DNA methylation change from pre- to post-treatment was not conditional on genotype (Supplementary Tables S3,S4).

In addition to analysing mean DNA methylation levels pre- and post-intervention for each gene, we also analysed DNA methylation levels of individual CpGs. Intervention-associated changes in DNA methylation levels of single CpGs did not survive correction for multiple testing (Supplementary Table S2).

### Study 2—Explorative whole-genome bisulfite sequencing

In addition to our goal of replicating previously reported results in candidate genes, we aimed to explore whether we could identify genomic regions with large pre-post intervention changes in DNA methylation, which could then be validated in the larger sample. Following recommendations by Ziller et al.^35^, we sequenced at least two biological replicates at > 5 × coverage. Pre- and post-intervention monocyte DNA from two patients with PTSD was subjected to WGBS. The patients for the WGBS were selected based on their responsiveness to therapy, absence of co-morbidity, and non-smoking status.

**Figure 3:**
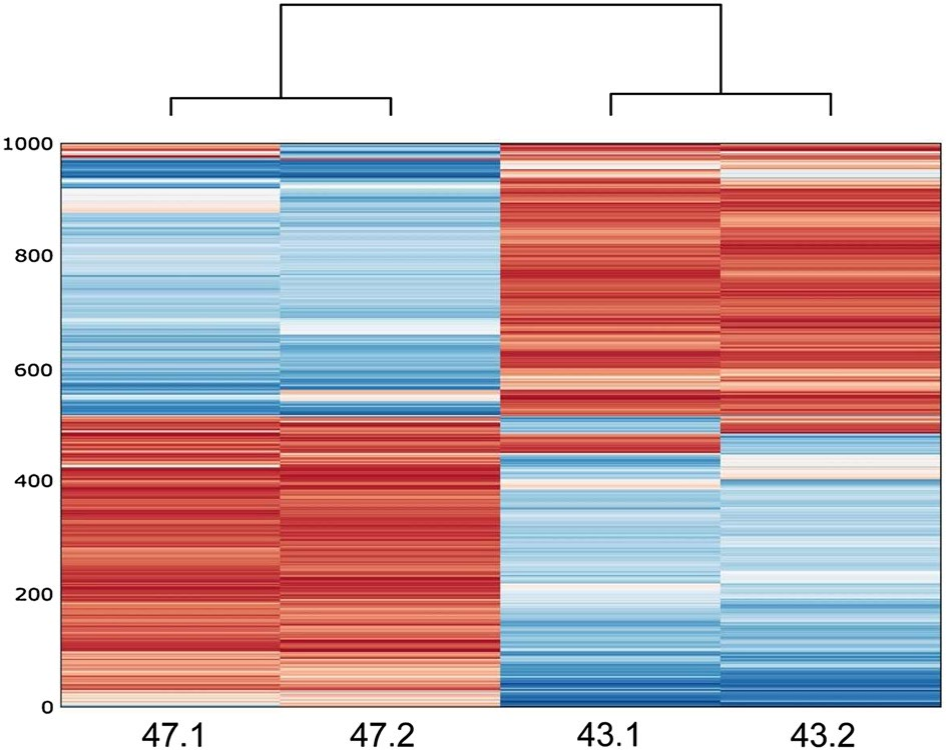
Cluster analysis of 1000 most variable CpGs. Clustered heatmap showing monocyte DNA methylation from two patients with PTSD (47 and 43), pre- (.1) and post-intervention (.2). CpG SNPs were excluded from the analysis. On the Y-axis, the 1,000 most variable CpGs are shown with DNA methylation levels ranging from blue (no methylation) to red (100% methylated).

Overall DNA methylation did not differ between pre- and post-intervention samples of the same individuals (p-value = 1; Wilcoxon signed rank test, Supplementary Table S5). A principal component analysis (PCA) of the PTSD methylomes did not group samples according to pre- or post-intervention status (Supplementary Fig. S3). The same was observed in a cluster analysis of the 1,000 most variable CpGs for the PTSD dataset, in which separate branches are observed for each individual (Fig. 3). We observed large differences between individuals, most likely reflecting the different genetic backgrounds, but no recurrent overall differences between the pre- and post-intervention statuses.

To identify DMRs between pre- and post-treatment, we used two different bioinformatic tools (camel and metilene^36,37^). We set the threshold to a minimum coverage of five reads. Defining a camel DMR as a region with at least four CpGs and a minimum DNA methylation difference of 0.3, we detected 33 DMRs in the PTSD patients (Supplementary Table S6). Using a filter of q < 0.05 for metilene DMRs, we detected four DMRs (Supplementary Table S7).

#### Validation of WGBS-nominated targets in the PTSD sample

DNA methylation patterns of four targets that emerged as potentially responsive to intervention from WGBS data were subject to validation in the entire sample (n = 60) using DBS. The following gene regions were analyzed: DMR-4 within *Adenosine A1 Receptor* (*ADORA1),* DMR-21 within *Tetraspanin 9 (TSPAN9),* DMR-10 within *Ribosomal Protein S6 Kinase A2 (RPS6KA2),* and intergenic DMR-1 (Supplementary Table S6). Chromosomal locations of the targets are shown in Supplementary Fig. S1.

The effect sizes of *ADORA1*, *RPS6KA2*, and DMR-1 were all significantly smaller than 5% DNA methylation change in both the whole cohort and the responder set (all p_corrected_ < 0.001). The effect of *TSPAN9* was only significantly different from 5% in the whole cohort on an uncorrected p = 0.043, which did not survive Bonferroni correction. There were no statistically significant differences in DNA methylation between pre- and post-intervention for the WGBS-nominated targets. However, the possibility of a true effect size within the range of 5% DNA methylation difference at the *TSPAN9* between pre- and post-intervention samples cannot be ruled out. Bayes factors favored the null hypothesis of no DNA methylation change for *RPS6KA2* and DMR-1. Similar to candidate genes, DNA methylation change was not conditional on therapy response (all p > 0.05; Table 1), with all Bayes factors larger than one and Bayes factors for DMR-1 above the threshold of three. There were no statistically significant correlations between symptom changes and DNA methylation changes (all p > 0.05; Table 2 and Supplementary Fig. S2, lower row), with all Bayes factors larger than one. Distribution of DNA methylation changes are shown in Fig. 1, lower row by responder status. The largest mean difference between time points was found for *TSPAN9* (− 2.78% ± 9.78%), and the smallest was − 0.12% ± 3.35% for DMR-1. Mean DNA methylation levels pre- and post-intervention averaged across the analyzed genomic regions are shown in Fig. 2. DNA methylation levels for single CpGs are shown in Supplementary Table S2.

Equivalence tests showed that the effect sizes of *ADORA1*, *RPS6KA2*, and DMR-1 were all significantly smaller than 5% DNA methylation change in both the whole cohort and the responder set (all p_corrected_ < 0.001). The effect of *TSPAN9* was only significantly different from 5% in the whole cohort on an uncorrected p = 0.043, which did not survive Bonferroni correction. Hence, although we cannot rule out that there is a true effect size as large as 5% DNA methylation change for the *TSPAN9* locus, we can conclude with relative certainty that true effect sizes of the WBGS nominated genes are smaller than 5%.

## Discussion

Epigenetic processes have been proposed as a potential mechanism mediating the link between exposure to trauma or adversity and mental health problems. Expectedly, there is great interest in the question whether psychological interventions translate into a modification of disorder-related epigenetic signatures. The first few studies addressing this question have found divergent patterns of DNA methylation changes between patients who responded to therapy and those who did not. However, results of these pilot studies remain inconclusive because differences in genetic background and changes in cell composition over time, which are the main confounders in epigenetic research, were largely not taken into account.

In our study, we avoided these confounders by using isogenic pre- and post-treatment samples and a homogenous cell population (monocytes). Furthermore, we used DBS for targeted high resolution DNA methylation analysis. With this approach, we could not identify any evidence for intervention-associated changes in DNA methylation. We investigated four candidate genes that were previously shown to be sensitive to variation in the social environment and/or seemed responsive to intervention in a sample of female PTSD patients. The prime candidate for epigenetic research in humans has been *NR3C1*, since DNA methylation of the promoter of alternative exon 1_7_ (1F in humans) had been shown to vary as a function of maternal care in the rodent model^6^. We found no changes in DNA methylation between pre- and post-intervention in PTSD patients and no differences between therapy responders and non-responders. We also investigated DNA methylation of *SLC6A4*, the most widely studied candidate gene in psychiatry. One previous study found small increases in *SLC6A4* DNA methylation in therapy responders in one of four assessed CpG sites^19^. Here, we investigated 82 CpGs sites across the entire CpG island in the gene’s promoter region and found no evidence for change over time, neither overall nor when stratified by responders and non-responders. This was also true for *FKBP5*, where we focused on 5 CpGs in intron 7, previously associated with PTSD risk following early life adversity^38^, and the *OXTR* gene, where we investigated 16 CpGs in a putative enhancer region—characterized by H3K27 acetylation peaks—in intron 3 of the gene. In summary, we did not observe intervention-associated changes in DNA methylation of commonly investigated candidate genes when assessed in a homogenous cell population, and we could not find any moderating effect of genotype on DNA methylation or treatment response in either *SLC6A4* or *FKBP5*. It is important to point out that we have not investigated all previously analyzed CpGs in mentioned candidate genes. Klengel and colleagues^38^ focused, beside *FKBP5* intron 7, on *FKBP5* intron 2 and we therefore cannot exclude any changes in this particular locus or other not analyzed loci.

In small samples with limited statistical power, non-significant results do not provide evidence for the absence of meaningful effects. We thus further conducted equivalence tests to ascertain whether observed effect sizes are significantly smaller than a minimal effect size of interest. These revealed that DNA methylation changes of the four candidate genes were significantly below physiologically meaningful levels^39,40^. Notably, this does not preclude that very small changes in these genes might contribute to a meaningful epigenome-wide poly-epigenetic score, but such studies necessitate much larger samples and another theoretical perspective on the importance of single candidate genes than the studies whose effects we aimed to replicate.

Candidate gene studies can only provide a very limited view on potential dynamics of the epigenome. True epigenome-wide studies covering the ~ 28 million CpG sites across the human genome are still prohibitive in large cohorts because of the associated costs. Here, we used an explorative strategy to identify genomic regions where DNA methylation patterns might potentially be responsive to intervention by using WGBS in two PTSD patients before and after therapy. We identified a small number of pre-to-post intervention DMRs (n = 36). The investigation of a subset of the identified PTSD DMRs in the remaining 58 patients could not confirm the potential differential DNA methylation patterns found by WGBS.

This suggests that cell type-specific DNA methylation patterns are highly stable. This stability can probably be attributed to stem or progenitor cells, because monocytes have a very short half-life in blood. Pre- and post-treatment samples are not drawn from the same pool of monocytes, but are derived from the same stem or progenitor cells. It is important to reflect on the question whether it is plausible to expect changes of DNA methylation patterns in peripheral surrogate tissues that are associated with changes in thoughts, feelings or somatic alterations in a meaningful way. Three scenarios have been put forward previously: first, DNA methylation dynamics observed in blood or buccal epithelial cells reflect the processes occurring in neuronal cells^20^. However, as buccal epithelium or leukocytes do not have a biologically realistic link to cellular processes occurring in neurons, these transient and activity-dependent alterations of chromatin and the specific patterns of DNA methylation in neurons are most likely not reflected in peripheral tissue.

The second scenario is that changes in peripheral DNA methylation patterns are brought about by intervention-associated psychophysiological changes that parallel changes in thoughts and feelings. Such cross talk between the central nervous system and peripheral organs is arguably mediated through regulation of the autonomic nervous system and the hypothalamic–pituitary–adrenal axis, activating the two main stress effectors noradrenaline and cortisol. These mediate their effects by engaging cellular receptor systems, which ultimately regulate specific gene expression responses. A testable model therefore suggests that changes in DNA methylation are brought about by alterations of transcriptional activity associated with changes in upstream signaling of stress mediators such as cortisol or noradrenaline^41^.

The third scenario, which is in our opinion the most plausible explanation for many previous findings, is that changes in DNA methylation levels merely reflect changes in cell composition, which might be stochastic in nature, reflect differences in health status independent of the disorder of interest or reflect intervention-associated physiological changes. Changes in cell composition were not assessed in the present study but might in principle be a marker of therapy response^42^.

An important limitation of this study is that the costs associated with WGBS limited the number of patients to 2, so that only large pre-to-post differences in this exploratory analysis could be reliably identified. Power estimates show that we had 80% power to detect pre-to-post-treatment DNA methylation differences of 30%, larger samples are needed to identify smaller changes. Furthermore, not all identified DMRs could be tested in the entire cohort. It cannot be ruled out that other, non-tested DMRs would have shown a DNA methylation change in the whole cohort. In the future, sufficiently powered longitudinal studies with repeated WGBS analyses across different timepoints would better address these questions.

Furthermore, only one cell type was investigated. Monocytes have been shown to demonstrate considerable sensitivity to social conditions and traumatic experiences, at least in terms of transcriptional changes^27,43–45^. However, any effects in other cell types (e.g., T cells, B cells, NK cells, etc.), or changes in the relative prevalence of cells in the circulating leukocyte pool are missed in this analysis. Finally, therapy-associated functional differences at the level of gene expression might have occurred independently of DNA methylation variation.

## Conclusions

In conclusion, our results provide no evidence in support of intervention-associated changes in DNA methylation in monocytes in PTSD, and equivalence tests and Bayesian statistics provided evidence against even subtle DNA methylation changes associated with therapy outcome. We argue that the previously reported changes in DNA methylation following therapy are most likely explained by changes in cell composition rather than by cellular reprogramming of DNA methylation. In principle, any shifts in cellular composition might reflect intervention-associated physiological changes and could therefore be used as biomarkers, but markers should not be confused with mechanisms.

## Methods

### Sample characteristics, clinical features and treatment

We investigated sixty female in-patients of European descent seeking treatment for PTSD at the Department of Psychosomatic Medicine and Psychotherapy, LWL-University Hospital, Ruhr-University Bochum. The patients were between 20 and 60 years old (mean age: 40.0 ± 11.9 (SD) years), and the mean treatment duration was 6.5 ± 1.4 (SD) weeks. Inclusion criteria were PTSD diagnosis and female sex. All patients with PTSD were diagnosed with ICD-10 (F43.1; International Classification of Diseases, 10th revision, WHO, 1993) prior to in-patient admission via structured clinical interviews in the outpatient department of the hospital. Furthermore, symptoms of PTSD were recorded before and after in-patient treatment with the PCL-5 (see Supplementary Table S8 for more details).

**Figure 4:**
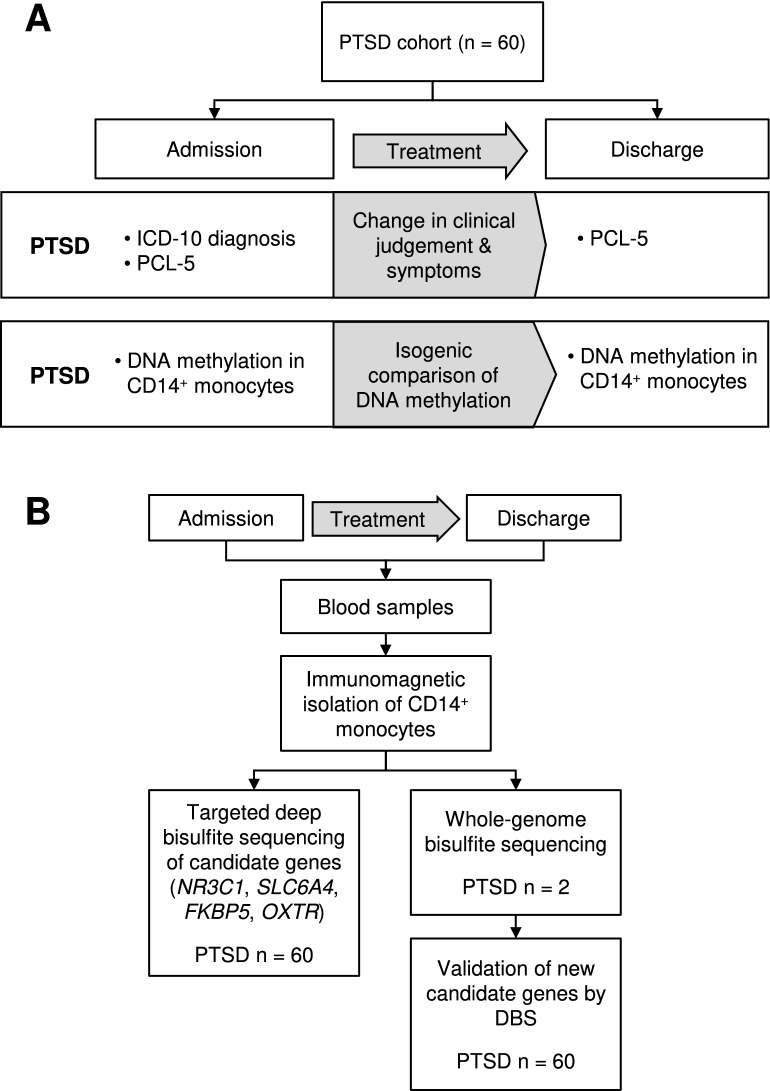
Overview of the study design. (**A**) Study design indicating diagnostic instruments and questionnaires and sample collection at admission and discharge; (**B**) Flow chart of the laboratory analysis procedure after sample collection.

Participants received standard in-patient PTSD treatment. This involved one session each week of individual cognitive-behavioral therapy, three sessions of trauma group therapy, two sessions of trauma stabilization group therapy, one session of a “skills group”, two sessions of kinesitherapy, two sessions of art therapy, physiotherapy, clinical rounds, and daily short sessions with a nurse. During individual therapy sessions patients received different trauma exposure methods. Overview of the study design is shown in Fig. 4.

### Ethics approval and consent to participate

The study was performed in accordance with the ethical standards of the Declaration of Helsinki (1964) and its subsequent amendments. The PTSD study was approved by the ethics committee of the Faculty of Psychology, Ruhr University Bochum (Nr. 155). Patients gave written informed consent.

### Sample preparation and genotyping

9 ml blood was drawn in the morning between 7 and 9 am (S-Monovette 9 ml K3E, Sarstedt). Monocytes were immunomagnetically purified from whole blood with the MACS System (Miltenyi Biotec), shock frozen and stored at − 80 °C. Cell homogeneity was determined with the BD FACSCanto TM II Flow Cytometer (BD Biosciences), and showed high purity (98.3 ± 2.4% (SD). DNA was isolated with the AllPrep RNA/DNA Mini Kit (Qiagen, 80204).

5-HTTLPR polymorphism was analyzed as previously described^46^*. FKBP5* genotype of rs1360780 was evaluated using high resolution melt analysis (HRM; Biorad). Further information can be found in Supplementary Table S10.

### Targeted deep bisulfite sequencing

For DBS, DNA was bisulfite-modified with the EZ DNA Methylation-Gold Kit (Zymo Research, D5005) and amplified in two rounds of PCR with specific primers, including adapters for sequencing, as described in Supplementary Table S9. After quality control and purification of the PCR products, the samples were quantified and adjusted for similar copy numbers for subsequent NGS analysis as described by Moser and colleagues^47^. The following paired-end sequencing was performed on a MiSeq system (Illumina) using the Illumina MiSeq reagent Kit v2 (500 cycles- 2 × 250 paired end) in collaboration with the ‘BioChip Labor' of the Center of Medical Biotechnology (ZMB, University Duisburg/Essen). The raw sequencing data was analyzed using the amplikyzer2^48^. The software enables demultiplexing of the sequencing data, creation of FASTQ files and the output of DNA methylation values in percent for each CpG.

### Whole-genome bisulfite sequencing

WGBS libraries were prepared as previously described^49^. Briefly, 20 ng DNA supplemented with 1% unmethylated lambda-DNA (Promega, D152A) were treated incubated in a 50-μl reaction with 1.6 μl of Tn5 transposase at 1 × TD buffer from the Nextera library preparation kit (Illumina, FC-121–1030) for 5 min at 55 °C. Following purification and gap repair, tagged DNA was bisulfite converted using the EZ DNA Methylation-Gold Kit (Zymo Research, D5005). Indexed-libraries were obtained by enrichment PCR in 40 μl reactions containing 1 × HotStarTaq Master Mix (Qiagen, 203445), 100 nM of each primer and 10 μl bisulfite-converted DNA (PCR settings: 95 °C 15 min, 12 × (95 °C 30 s, 53 °C 2 min, 72 °C 1 min and 72 °C 7 min). Reactions were purified twice using 0.8 × volume AMPure XP Beads (Beckman Coulter, A63881) and eluted in 10 μl EB buffer (Qiagen, 19086). Libraries were sequenced in HiSeq4000 75-bp paired-end runs (Illumina) using one lane per sample. Read data were processed as described previously^50,51^. In brief, the reads were aligned against hg19 using bwa-meth v0.2.0^52^, sorted by samtools v1.3.1^53^, deduplicated by sambamba^54^ v0.6.0 and their quality controlled by qualimap v2.2.2^55^. Camel v1.0^37^ was used to call DNA methylation levels and generate DMRs with a threshold of four CpGs, a minimum coverage of five and a minimum DNA methylation difference of 0.3. Additional DMRs were called by metilene v0.2.3^36^ and default parameters and a threshold of q < 0.05. Statistical downstream analysis were performed with Scipy^56^ and plots generated by matplotlib^57^.

### Statistical analyses

Repeated measures analyses of variance (ANOVA) were performed to assess changes in DNA methylation levels. Therapy response was included as an additional between-subject factor to check for therapy outcome-dependent changes in DNA methylation. Moreover, Pearson correlations were computed between percent DNA methylation change and PCL-5 symptom change to test a continuous measure of therapy response. In addition to changes over time, linear regression analysis was performed with pre-treatment mean DNA methylation of DBS targets and severity of baseline PTSD symptoms. We observed slight departures from normality, with six out of thirty-two ANOVA cells having an absolute skew greater than one. The maximum absolute skew of 1.42 was observed for *OXTR* at the second time point in the responder group. To test for the robustness of our results against violations of the normality assumption, we conducted permutation tests using the ez package (v4.4.0) and 1000 permutations. The p-values were overall consistent with the parametric results (Supplementary Table S11). Two notable differences were that the p-values for the main effect of time decreased from 0.065 to 0.035 for *ADORA1* and from 0.078 to 0.050 for *TSPAN9*. Neither are statistically significant after correction for multiple comparisons. Departures from normality were smaller for the DNA methylation change scores between pre- and post-measurement. P-values for their correlations with PCL-5 change scores did not differ substantially when performing a non-parametric permutation test (Supplementary Table S11).

Our sample size of *N* = 60 was based on the average sample size of previous studies in therapy-contingent DNA methylation changes in mental disorders (*M* = 69, range = 16–115). Due to the heterogeneity of reported effect sizes, statistical tests, designs, and populations, an effect size estimation based on previous studies was not suitable in our case. We thus performed a sensitivity analysis with the software *gpower*^58^*,* which showed acceptable statistical power (β = 0.80) to detect small changes from pre- to post-treatment at η^2^ = 0.03, assuming a correlation between pre- and post-intervention DNA methylation of *r* = 0.50. For comparisons between responders and non-responders or correlations between symptom change and DNA methylation change, moderate effect sizes at η^2^ = 0.09 can be detected with sufficient statistical power, which is still in the range of previously reported effect sizes^15^.

Generally, non-significant results do not provide evidence for the null hypothesis (i.e. absence of effect), especially when the statistical power is limited. Therefore, p-values from mean DNA methylation change per analyzed gene were supplemented with Bayes factors to quantify the evidence for the null hypothesis using the BayesFactor package (v0.9.12-4.2) in R (3.6.1) and non-informative default priors^59,60^.

Complementary to the Bayesian approach, we conducted equivalence tests to assess whether effect sizes in mean DNA methylation are significantly smaller than the smallest biologically meaningful effect size. It has been argued that DNA methylation below 5% should be interpreted with extreme caution^39,40^. We used the two one-tailed t-test procedure^61^ to check whether empirical effect sizes for DNA methylation change are smaller than 5% or even a more conservative 1%. For a detailed account of statistical methods please see Supplementary Methods S1.

### Preprint

The preprint version of this article is present on https://www.medrxiv.org/con-tent/10.1101/2020.11.11.20229567v2.

## Supplementary Information

Supplementary Information.

## Data Availability

The WGBS datasets generated and analyzed during the current study are available in the European Nucleotide Archive (ENA) under the accession number PRJEB38906. DBS data and analysis script to reproduce the statistical analyses can be found on the open science framework: https://osf.io/eagjx/?view_only=9737755805f045edaa1d757cc3e9ba84.
