# Supplementary material for: No evidence for intervention-associated DNA methylation changes in monocytes of patients with posttraumatic stress disorder
Source: Sci Rep. 2022 Oct 17;12:17347. doi: 10.1038/s41598-022-22177-1 (PMC9576776; doi:10.1038/s41598-022-22177-1)
Supplement: Supplementary file 1 — Supplementary Information. [file 41598_2022_22177_MOESM1_ESM.pdf]

Supplementary Information

1. Supplementary Tables 2

1.1 Table S1. Linear regression with pre-treatment mean DNA methylation and severity of baseline PTSD symptoms 2

1.2 Table S2. Mean DNA methylation levels at single CpG sites 3

1.3 Table S3. Genotype 7

1.4 Table S4. Effect of intervention by genotype and the effect of intervention by genotype by responder status interaction on DNA methylation 8

1.5 Table S5. WGBS descriptive information 9

1.6 Table S6. DMRs in the PTSD cohort identified with camel 10

1.7 Table S7. DMRs in the PTSD cohort identified with metilene 11

1.8 Table S8. Sample Characteristics 12

1.9 Table S9. Primer Sequences 13

1.10 Table S10. Genotyping 15

1.11 Table S11. Permutation tests 16

2. Supplementary Figures 17

2.1 Figure S1. Chromosomal location of analyzed regions of the PTSD cohort 17

2.1.1 *NR3C1* 17

2.1.2 *SLC6A4* 17

2.1.3 *OXTR* 18

2.1.4 *FKBP5* 18

2.1.5 *ADORA1* 19

2.1.6 *TSPAN9* 19

2.1.7 *RPS6KA2* 20

2.1.8 DMR-1 20

2.2 Figure S2. Correlations between symptom change and DNA methylation change in the PTSD cohort 21

2.3 Figure S3. Principal component analysis 22

3. Supplementary Methods 23

3.1 Methods S1. Statistical analysis 23

1. Supplementary Tables

1.1 Table S1. Linear regression with pre-treatment mean DNA methylation and severity of baseline PTSD symptoms

A) Candidate Genes	Variable	B	*β*	s.e.	p
NR3C1	α	0.010		0.001	
	PCL-pre	-4.907×10^{-6}	-0.032	0.000	0.820
	R^2	0.001			
	corr. R^2	-0.018			
	F(df=1;52)	0.053			
SLC6A4	α	0.061		0.005	
	PCL-pre	7.945×10^{-5}	0.119	0.000	0.376
	R^2	0.014			
	corr. R^2	-0.004			
	F(df=1;56)	0.798			
OXTR	α	0.908		0.010	
	PCL-pre	0.000	0.187	0.000	0.160
	R^2	0.035			
	corr. R^2	0.018			
	F(df=1;56)	2.023			
FKBP5	α	0.875		0.010	
	PCL-pre	0.000	0.208	0.000	0.118
	R^2	0.043			
	corr. R^2	0.026			
	F(df=1;56)	2.523			
**B) New Targets from WGBS**	Variable	**B**	***β***	**s.e.**	**p**
ADORA1	α	0.442		0.040	
	PCL-pre	-0.001	-0.133	0.001	0.319
	R^2	0.018			
	corr. R^2	0.000			
	F(df=1;56)	1.010			
TSPAN9	α	0.508		0.083	
	PCL-pre	0.002	0.145	0.001	0.279
	R^2	0.021			
	corr. R^2	0.003			
	F(df=1;56)	1.196			
RSP6KA2	α	0.499		0.046	
	PCL-pre	0.000	-0.018	0.001	0.895
	R^2	0.000			
	corr. R^2	-0.018			
	F(df=1;55)	0.018			
**DMR-1**	α	0.714		0.044	
	PCL-pre	0.000	0.043	0.001	0.746
	R^2	0.002			
	corr. R^2	-0.016			
	F(df=1;56)	0.106			

1.2 Table S2. Mean DNA methylation levels at single CpG sites

A) Candidate Genes

NR3C1 Location: chr5:142783911-142783541

CpG No.	Pre Intervention	Post Intervention	Difference	SD	$F_{(df=1;55)}$	*p*
CpG_1	0.66%	0.54%	-0.13%	0.95%	.960	.331
CpG_2	1.05%	0.89%	-0.16%	1.01%	1.432	.237
CpG_3	1.02%	1.05%	0.04%	1.26%	.045	.833
CpG_4	0.70%	1.00%	0.30%	1.19%	3.646	.061
CpG_5	0.64%	0.82%	0.18%	1.39%	.925	.340
CpG_6	0.96%	0.70%	-0.27%	1.31%	2.327	.133
CpG_7	0.80%	1.02%	0.21%	1.38%	1.341	.252
CpG_8	0.48%	0.50%	0.02%	0.92%	.021	.886
CpG_9	0.59%	0.55%	-0.04%	0.93%	.082	.776
CpG_10	0.88%	0.73%	-0.14%	1.14%	.887	.350
CpG_11	0.71%	0.55%	-0.16%	1.02%	1.382	.245
CpG_12	0.68%	0.93%	0.25%	1.30%	2.081	.155
CpG_13	1.13%	1.36%	0.23%	1.61%	1.169	.284
CpG_14	0.66%	0.61%	-0.05%	1.13%	.125	.725
CpG_15	0.79%	0.61%	-0.18%	1.28%	1.089	.301
CpG_16	0.68%	0.50%	-0.18%	0.94%	2.037	.159
CpG_17	0.95%	1.18%	0.23%	1.29%	1.805	.185
CpG_18	0.98%	1.13%	0.14%	1.27%	.707	.404
CpG_19	0.86%	0.93%	0.07%	1.20%	.197	.659
CpG_20	0.75%	0.73%	-0.02%	0.92%	.021	.886
CpG_21	0.66%	0.57%	-0.09%	1.08%	.380	.540
CpG_22	1.09%	1.07%	-0.02%	1.38%	.009	.923
CpG_23	0.96%	0.73%	-0.23%	1.06%	2.678	.107
CpG_24	1.21%	1.41%	0.20%	1.18%	1.547	.219
CpG_25	1.63%	1.11%	-0.52%	1.43%	7.376	.009
CpG_26	1.11%	1.20%	0.09%	1.32%	.254	.616
CpG_27	1.20%	1.34%	0.14%	1.46%	.538	.466
CpG_28	1.05%	0.98%	-0.07%	1.22%	.192	.663
CpG_29	1.02%	0.91%	-0.11%	1.07%	.558	.458
CpG_30	0.88%	0.80%	-0.07%	1.06%	.255	.616
CpG_31	1.36%	1.02%	-0.34%	1.63%	2.419	.126
CpG_32	0.80%	0.59%	-0.21%	1.19%	1.827	.182
CpG_33	0.66%	0.70%	0.04%	0.99%	.073	.788
CpG_34	1.30%	1.32%	0.02%	1.48%	.008	.929
CpG_35	0.59%	0.66%	0.07%	1.02%	.272	.604
CpG_36	0.64%	0.73%	0.09%	1.25%	.284	.596
CpG_37	1.46%	1.34%	-0.13%	1.27%	.546	.463
CpG_38	1.13%	1.04%	-0.09%	1.35%	.244	.623
CpG_39	2.05%	1.96%	-0.09%	1.82%	.135	.715
CpG_40	1.61%	1.36%	-0.25%	1.28%	2.127	.150
CpG_41	1.04%	1.14%	0.11%	1.29%	.387	.536
CpG_42	0.91%	1.21%	0.30%	1.19%	3.646	.061

SLC6A4 Location: chr17:28563283-28562328

CpG No.	Pre Intervention	Post Intervention	Difference	SD	$F_{(df=1;59)}$	*p*
CpG_03	5.23%	5.32%	0.08%	2.59%	.062	.804
CpG_02	1.98%	2.27%	0.28%	1.38%	2.533	.117
CpG_01	2.38%	2.48%	0.10%	1.35%	.330	.568

CpG_1	4.62%	4.72%	0.10%	2.75%	.079	.779
CpG_2	1.62%	1.80%	0.18%	1.24%	1.308	.257
CpG_3	1.73%	1.58%	-0.15%	1.33%	.768	.384
CpG_4	3.00%	2.70%	-0.30%	1.77%	1.726	.194
CpG_5	1.00%	0.80%	-0.20%	0.94%	2.744	.103
CpG_6	0.85%	0.85%	0.00%	0.97%	0.000	1.000
CpG_7	1.48%	1.30%	-0.18%	1.32%	1.155	.287
CpG_8	1.57%	1.63%	0.07%	1.34%	.149	.701
CpG_9	1.73%	1.72%	-0.02%	1.14%	.013	.910
CpG_10	2.45%	2.50%	0.05%	1.63%	.056	.813
CpG_11	2.12%	2.35%	0.23%	1.78%	1.032	.314
CpG_12	2.85%	2.68%	-0.17%	1.62%	.637	.428
CpG_13	1.43%	1.52%	0.08%	1.18%	.298	.587
CpG_14	2.03%	2.15%	0.12%	1.62%	.313	.578
CpG_15	2.42%	2.23%	-0.18%	1.72%	.680	.413
CpG_16	1.72%	2.02%	0.30%	1.75%	1.764	.189
CpG_17	3.53%	4.08%	0.55%	2.00%	4.521	.038
CpG_18	1.37%	1.58%	0.22%	1.62%	1.078	.303
CpG_19	1.58%	1.82%	0.23%	2.68%	.454	.503
CpG_20	1.07%	1.33%	0.27%	2.28%	.818	.369
CpG_21	1.02%	1.05%	0.03%	1.19%	.047	.829
CpG_22	1.85%	1.83%	-0.02%	1.66%	.006	.938
CpG_23	2.15%	2.12%	-0.03%	1.33%	.038	.846
CpG_24	1.90%	1.68%	-0.22%	1.60%	1.107	.297
CpG_25	2.92%	2.70%	-0.22%	1.73%	.943	.335
CpG_26	2.02%	1.87%	-0.15%	1.61%	.518	.474
CpG_27	2.92%	2.67%	-0.25%	2.04%	.141	.709
CpG_28	1.98%	1.82%	-0.17%	1.42%	.831	.366
CpG_29	5.63%	5.95%	0.32%	2.43%	1.023	.316
CpG_30	2.62%	2.02%	-0.60%	1.71%	7.392	.009
CpG_31	2.08%	1.77%	-0.32%	1.51%	2.630	.110
CpG_32	3.53%	3.00%	-0.53%	2.37%	3.043	.086
CpG_33	1.55%	1.70%	0.15%	1.67%	.487	.488
CpG_34	1.62%	1.85%	0.23%	1.38%	1.710	.196
CpG_35	2.08%	1.98%	-0.10%	1.72%	.202	.655
CpG_36	3.23%	2.70%	-0.53%	1.88%	4.819	.032
CpG_37	2.27%	2.32%	0.05%	1.60%	.059	.809
CpG_38	1.67%	1.75%	0.08%	1.32%	.240	.626
CpG_39	2.80%	2.45%	-0.35%	1.69%	2.587	.113
CpG_40	3.15%	2.83%	-0.32%	2.13%	1.330	.254
CpG_41	1.25%	1.07%	-0.18%	1.10%	1.676	.200
CpG_42	1.38%	1.38%	0.00%	1.18%	0.000	1.000
CpG_43	1.73%	1.83%	0.10%	1.54%	.254	.616
CpG_44	1.90%	1.73%	-0.17%	1.39%	.860	.358
CpG_45	2.45%	2.42%	-0.03%	1.51%	.029	.865
CpG_46	3.77%	3.88%	0.12%	1.97%	.211	.648
CpG_47	3.23%	3.08%	-0.15%	1.91%	.369	.546
CpG_48	6.42%	6.10%	-0.32%	2.75%	.794	.376
CpG_49	7.67%	7.40%	-0.27%	3.19%	.418	.520
CpG_50	10.15%	10.48%	0.33%	3.29%	.617	.435
CpG_51	9.92%	9.52%	-0.40%	3.93%	.622	.433
CpG_52	5.25%	5.05%	-0.20%	2.20%	.496	.484
CpG_53	10.97%	10.32%	-0.65%	3.37%	2.227	.141
CpG_54	13.27%	13.43%	0.17%	3.52%	.135	.715
CpG_55	19.32%	18.52%	-0.80%	4.67%	1.760	.190
CpG_56	5.23%	5.37%	0.13%	2.76%	.140	.709
CpG_57	10.62%	10.95%	0.33%	4.10%	.397	.531
CpG_58	12.45%	11.92%	-0.53%	4.38%	.889	.350
CpG_59	13.63%	13.85%	0.22%	3.33%	.253	.617
CpG_60	14.37%	14.57%	0.20%	4.28%	.131	.719

CpG_61	3.58%	3.60%	0.02%	2.13%	.004	.952
CpG_62	1.08%	1.20%	0.12%	1.11%	.668	.417
CpG_63	3.65%	4.05%	0.40%	2.37%	1.704	.197
CpG_64	3.67%	3.55%	-0.12%	2.20%	.168	.683
CpG_65	3.93%	4.48%	0.55%	2.59%	2.698	.106
CpG_66	18.27%	19.23%	0.97%	3.88%	3.717	.059
CpG_67	24.67%	24.23%	-0.43%	5.26%	.407	.526
CpG_69	29.72%	31.30%	1.58%	5.55%	4.885	.031
CpG_70	12.17%	13.20%	1.03%	2.80%	8.183	.006
CpG_71	13.80%	14.07%	0.27%	3.75%	.304	.583
CpG_72	10.23%	9.63%	-0.60%	3.51%	1.750	.191
CpG_73	10.67%	10.77%	0.10%	3.74%	.043	.836
CpG_74	19.82%	20.20%	0.38%	5.19%	.327	.570
CpG_75	21.38%	21.50%	0.12%	5.17%	.031	.862
CpG_77	11.90%	12.68%	0.78%	3.72%	2.661	.108
CpG_78	8.12%	8.70%	0.58%	2.92%	2.397	.127
CpG_79	12.87%	14.03%	1.17%	3.84%	5.549	.022
CpG_80	29.75%	30.80%	1.05%	4.97%	2.679	.107
CpG_81	41.18%	41.90%	0.72%	5.62%	.975	.327

***OXTR* Enhancer** Location: chr3:8798082-8800976

CpG No.	Pre Intervention	Post Intervention	Difference	SD	$F_{(df=1;59)}$	*p*
CpG_5	93.15%	93.38%	0.23%	4.14%	.190	.664
CpG_6	95.72%	95.82%	0.10%	7.10%	.012	.913
CpG_7	86.30%	84.83%	-1.47%	6.66%	2.912	.093
CpG_8	95.37%	95.82%	0.45%	3.18%	1.205	.277
CpG_9	88.67%	88.45%	-0.22%	5.73%	.086	.771
CpG_24	93.00%	93.68%	0.68%	4.79%	1.222	.274
CpG_25	82.98%	81.70%	-1.28%	5.97%	2.776	.101
CpG_26	94.02%	93.07%	-0.95%	3.65%	4.071	.048
CpG_28	96.50%	96.40%	-0.10%	2.67%	.084	.772
CpG_29	93.08%	93.53%	0.45%	3.06%	1.297	.259
CpG_30	96.62%	96.42%	-0.20%	2.18%	.506	.479
CpG_31	92.45%	93.30%	0.85%	3.88%	2.875	.095
CpG_32	95.37%	94.78%	-0.58%	3.57%	1.601	.211
CpG_33	89.87%	89.05%	-0.82%	5.32%	1.416	.239
CpG_34	90.42%	90.12%	-0.30%	3.78%	.378	.541
CpG_35	90.80%	89.75%	-1.05%	6.45%	1.591	.212

FKBP5 Location: chr6:35558322-35558593

CpG No.	Pre Intervention	Post Intervention	Difference	SD	$F_{(df=1;59)}$	*p*
CpG_1	78.93%	79.12%	0.18%	2.59%	.300	.586
CpG_2	97.15%	97.23%	0.08%	1.00%	.420	.520
CpG_3	97.83%	97.95%	0.12%	1.15%	.616	.436
CpG_4	80.98%	81.05%	0.07%	2.65%	.038	.846
CpG_5	89.92%	90.07%	0.15%	2.72%	.183	.671

B) New Targets from WGBS

ADORA1 Location: chr1:203097582-203097833

CpG No.	Pre Intervention	Post Intervention	Difference	SD	$F_{(df=1;59)}$	*p*
CpG_1	19.57%	18.80%	-0.77%	4.64%	1.637	.206

CpG_2	53.35%	53.52%	0.17%	5.25%	.061	.807
CpG_3	40.30%	42.53%	2.23%	9.15%	3.574	.064
CpG_4	41.62%	42.67%	1.05%	8.27%	.967	.329
CpG_5	48.85%	48.55%	-0.30%	7.95%	.085	.771
CpG_6	26.32%	27.92%	1.60%	6.74%	3.378	.071
CpG_7	52.08%	53.97%	1.88%	8.87%	2.704	.105

TSPAN9 Location: chr12:3370787-3371092

CpG No.	Pre Intervention	Post Intervention	Difference	SD	$F_{(df=1;59)}$	*p*
CpG_1	60.28%	55.57%	-4.72%	18.91%	3.732	.058
CpG_2	83.42%	82.65%	-0.77%	19.53%	.092	.762
CpG_3	52.83%	51.33%	-1.50%	17.32%	.450	.505
CpG_4	43.80%	40.70%	-3.10%	17.02%	1.991	.163
CpG_5	63.57%	59.93%	-3.63%	16.11%	3.053	.086
CpG_6	54.73%	51.73%	-3.00%	15.50%	2.249	.139

RPS6KA2 Location: chr6:166997045-166996725

CpG No.	Pre Intervention	Post Intervention	Difference	SD	$F_{(df=1;58)}$	*p*
CpG_1	79.80%	81.19%	1.39%	6.56%	2.646	.109
CpG_2	56.85%	57.17%	0.32%	7.14%	.120	.730
CpG_3	67.66%	68.07%	0.41%	6.66%	.220	.641
CpG_4	36.81%	36.31%	-0.51%	7.23%	.292	.591
CpG_5	23.90%	23.53%	-0.37%	6.90%	.173	.679
CpG_6	52.39%	52.56%	0.17%	6.05%	.046	.831
CpG_7	38.56%	37.71%	-0.85%	7.11%	.837	.364
CpG_8	38.15%	37.31%	-0.85%	7.63%	.728	.397

DMR-1 Location: chr1:149233322-149233624

CpG No.	Pre Intervention	Post Intervention	Difference	SD	$F_{(df=1;59)}$	*p*
CpG_2	54.63%	55.15%	0.52%	6.19%	.418	.521
CpG_3	43.47%	43.05%	-0.42%	5.57%	.336	.564
CpG_4	64.43%	63.37%	-1.07%	6.22%	1.765	.189
CpG_6	68.55%	68.70%	0.15%	6.14%	.036	.850
CpG_7	64.05%	64.02%	-0.03%	6.63%	.002	.969
CpG_8	75.40%	74.70%	-0.70%	5.81%	.871	.355
CpG_9	82.80%	82.72%	-0.08%	4.80%	.018	.893
CpG_10	57.87%	57.67%	-0.20%	7.18%	.047	.830
CpG_11	78.22%	77.47%	-0.75%	5.54%	1.102	.298
CpG_12	77.18%	77.53%	0.35%	5.93%	.209	.649
CpG_13	81.43%	81.75%	0.32%	5.13%	.229	.634
CpG_14	87.35%	87.50%	0.15%	4.99%	.054	.817
CpG_15	90.60%	90.33%	-0.27%	3.80%	.296	.589
CpG_16	92.42%	92.73%	0.32%	3.72%	.436	.512

1.3 Table S3. Genotype

Gene	Genotype	N	%
5-HTTLPR (*SLC6A4*)	l/l	20	33,3
	s/l	29	48,3
	s/s	11	18,3
rs1360780 (*FKBP5*)	c/c	31	51,7
	c/t	24	40,0
	t/t	5	8,3

1.4 Table S4. Effect of *intervention* by *genotype* and the effect of *intervention* by *genotype* by *responder* status interaction on DNA methylation

	Genotype x Intervention			Genotype x Intervention x Responder		
	F	*p*	η_G^2	*F*	*p*	η_G^2
SLC6A4	1.096	.342	.041	1.425	.250	.053
FKBP5	0.000	.992	.000	0.011	.917	.000

Note. Results from a mixed model analysis of variance. η_G^2 = generalized eta squared. Numerator *df* for *SLC6A4*= 2 and for *FKBP5* = 1. Denominator *df* for *SLC6A4*= 51 and for *FKBP5* = 53. *FKBP5:* Thirty-one patients were homozygotes for the C allele, twenty-four were CT heterozygotes, and five were homozygotes for the T allele. Because homozygous TT samples were rare in our sample, we grouped them with heterozygous CT samples for analysis.

1.5 Table S5. WGBS descriptive information

Cohort	Sample (ENA)	before/after	Conversion rate	Mapping efficiency	Duplication Rate	Coverage	Mean methylation
PTSD	47.1 (K002000217_85588)	before	1	1	0.21	9.11	0.72
PTSD	47.2 (K002000217_85589)	after	1	1	0.21	8.33	0.72
PTSD	43.1(K002000217_85590)	before	1	1	0.22	8.19	0.72
PTSD	43.2 (K002000217_85591)	after	1	1	0.22	8.07	0.72

1.6 Table S6. DMRs in the PTSD cohort identified with camel

#	chrom	start	stop	cpg	mean_meth_diff	gene	cgi	overlap metilene	DBS
1	1	149233364	149233394	4	0.31				yes
2	1	229119599	229120672	8	0.30				
3	1	3685641	3685665	4	-0.30	*CCDC27*			
4	1	203097628	203097776	7	-0.32	*ADORA1*			yes
5	2	178034563	178034833	5	0.30				
6	2	88302827	88302847	4	-0.40				
7	2	104445362	104447294	8	-0.37				
8	4	132984311	132984336	4	-0.30				
9	5	4512352	4512371	4	0.32				
10	6	166996807	166996856	4	0.31	*RPS6KA2*			yes
11	6	28863695	28863746	4	-0.41			yes	
12	7	1595247	1595280	4	0.34	*TMEM184A*			
13	7	101514184	101514315	5	0.31	*CUX1*			
14	7	148768873	148768932	7	-0.39	*ZNF786*	yes		
15	8	35549566	35549812	4	0.31	*UNC5D*			
16	8	141109357	141109414	5	0.32	*TRAPPC9*	yes		
17	10	131052326	131052347	4	-0.31				
18	11	1892538	1892592	4	0.38	*LSP1*	yes		
19	11	45951791	45951817	4	-0.33	*PHF21A*			
20	12	130615735	130615819	4	0.31				
21	12	3370834	3371031	6	-0.36	*TSPAN9*			yes
22	12	133181087	133181120	4	-0.40	*LRCOL1*			
23	13	41496184	41496213	5	-0.33		yes		
24	15	88874573	88874627	4	-0.33				
25	16	75031951	75032022	7	0.30				
26	16	81924284	81924435	5	0.38	*PLCG2*			
27	20	57426931	57427018	11	0.30	*GNAS*			
28	20	57430980	57431013	4	-0.38	*GNAS*	yes		
29	22	19086801	19086831	4	0.35	*DGCR2*			
30	X	74144931	74144954	4	0.37	*KIAA2022*	yes		
31	X	102879244	102879668	6	0.37				
32	X	119149621	119149673	8	0.35		yes		
33	X	34836096	34836268	4	-0.30				

1.7 Table S7. DMRs in the PTSD cohort identified with metilene

#	#chr	start	stop	q-value	mean methylation difference	#CpGs	p (MWU)	p (2D KS)	mean g1	mean g2	cgi	gene	overlap camel
1	6	28863663	28863803	0.0270	36.36	11	1.30E-08	3.90E-09	55.682	19.318			yes
2	6	32847513	32847758	0.0450	33.72	16	1.40E-10	6.70E-09	86.875	53.156	yes		
3	8	105379654	105379756	0.0140	39.54	12	3.70E-09	2.10E-09	72.417	32.875	yes		
4	9	140312096	140312262	0.0037	31.76	25	1.30E-13	5.40E-10	83.760	52.000	yes	*EXD3*	

1.8 Table S8. Sample Characteristics

PTBS cohort	N	Min	Max	Mean	SD
**Age [years]**	60	20	60	40	11.86
**BMI [kg/m²]**	60	17.8	45.5	30.1	7.26
**treatment duration [days]**	60	20	69	45.35	9.87
**PTSD symptoms pre (PCL-5)**	58	22	77	55.87	11.53
**PTSD symptoms post (PCL-5)**	59	5	80	40.37	16.92
**Leukocytes pre [cells/nl]**	59	3.5	16.3	7.8	2.52
**Leukocytes post [cells/nl]**	54	3.7	13.1	7.3	2.03
**Platelets pre [cells/nl]**	59	125	524	282.85	79.72
**Platelets post [cells/nl]**	57	172	469	284.37	76.43
		**Yes**	**No**		
**Smoking**	59	33	26		
**psychotropic medication (pre)**	57	48	9		
**psychotropic medication (post)**	60	56	4		
**Diagnosis of PTSD**	60	60	0		
**Diagnosis of depression**	60	57	3		
**other psychological comorbidities**	60	31	29		
**somatic comorbidities**	60	26	34		

1.9 Table S9. Primer Sequences

All genome positions given correspond to hg19/GRCh37.

PCRs in the AN cohort were performed as described in Leitão et al. 2018. PCRs in the PTSD cohort are described below.

1.PCR: The first round PCR reaction contained 1 µl of bisulfite-converted DNA. 0.125 µM primers and 5.5 µl GoTaq® G2 Hot Start Master Mixes (Promega. Fitchburg. WI. USA) in a total volume of 11 µl. The standard amplification protocol included an initial denaturation step for 2 min at 95 °C. followed by 50 cycles of melting at 94 °C for 30 s. annealing at 54.7-61.5 °C for 45 s and extension at 72 °C for 45 s. Followed by an additional extension at 72 °C for 10 min at the end of the 50 cycles. All PCRs included nontemplate controls. The primer sequences are depicted in the table. The red sequence is a tag and used as a template for the second round PCR.

DMR/ Gene	Chromosomal location	Primer	Sequence 5´-3´	Length Target [bp]	Annealing Temp. [°C]	Analyzed CpGs
**PTSD cohort**						
**A) Candidate Genes**						
NR3C1	chr5:142.783.541-142.783.911	forward	CTTGCTTCCTGGCACGAGGGGGGTAGATTTGGTTTTTT	371	56.9	42
		reverse	CAGGAAACAGCTATGACTCCCTTCCCTAAAACCTC			
SLC6A4 Promoter	chr17:28.562.939-28.563.283	forward	CTTGCTTCCTGGCACGAGTAGGAGGGGAGGGATTTT	345	59.4	23
		reverse	CAGGAAACAGCTATGACAAACCTCTAAACTAAACTCACATC			
	chr17:28.562.574-28.562.952	forward	CTTGCTTCCTGGCACGAGGGGAAGAAGGTTTGGAAAGA	379	59.4	42
		reverse	CAGGAAACAGCTATGACTCCCTCCCCTCCTAACTCTAA			
	chr17:28.562.328-28.562.682	forward	CTTGCTTCCTGGCACGAGTTTTTAAGGGTTTTTAAGAGGTTGTAAAGT	355	60.0	17
		reverse	CAGGAAACAGCTATGACAAACCAACCCCCCTACCCAACCC			
OXTR Enhancer	chr3:8.799.262-8.799.615	forward	CTTGCTTCCTGGCACGAGTTGTGGGTAGGAGTAGGATTTTA	354	59.4	5
		reverse	CAGGAAACAGCTATGACTTCTCATCCTAAATCTAAAAATCACTT			
	chr3:8.800.371-8.800.739	forward	CTTGCTTCCTGGCACGAGAGTGTAAGGTTTGGGTGAA	369	54.7	11
		reverse	CAGGAAACAGCTATGACATCTAAAATAAAACCCCAAAAATT			
FKBP5	chr6:35.558.322-35.558.593 272	forward	CTTGCTTCCTGGCACGAGTTTTGGGTTGAGGATAGAAAGG	272	56	5
		reverse	CAGGAAACAGCTATGACATCCAAAACAACTAACAAATTCTCT			
**B) New Targets from WGBS**						
DMR-4 *ADORA1*	chr1:203.097.582-203.097.833	forward	CTTGCTTCCTGGCACGAGTTGTGTATAGGGGTGGGTAGA	252	61.5	7
		reverse	CAGGAAACAGCTATGACACCCTATAATATATCCCACTTATCAC			
DMR-21 *TSPAN9*	chr12:3.370.787-3.371.092	forward	CTTGCTTCCTGGCACGAGAATGGGGATGTTTAGTTAGTGTTATTTAG	306	61.5	6
		reverse	CAGGAAACAGCTATGACACACACAAAAAATAAACCTACTACTTTTC			

DMR-10 *RPS6KA2*	chr6:166.996.725-166.997.045	forward	CTTGCTTCCTGGCACGAGTGGGGAATTTTGGTGTAGATATGA	321	61.5	8
		reverse	CAGGAAACAGCTATGACCACTACCTCATCCAAAACCCTAAT			
DMR-1	chr1:149.233.322-149.233.624	forward	CTTGCTTCCTGGCACGAGTGAAGTTTTTTTGTAGGTTATAGGGAAGGG	303	61.5	14
		reverse	CAGGAAACAGCTATGACCTACCCAATCTTTCTCTTTCTTAAT			

Length of the amplicons is stated without tags.

2.PCR: The second round PCR reaction contained 1 µl of PCR product of the first round. 0.2 µM primers and 5 µl GoTaq® G2 Hot Start Master Mixes (Promega. Fitchburg. WI. USA) in a total volume of 10 µl. The standard amplification protocol included an initial denaturation step for 2 min at 95 °C. followed by 35 cycles of melting at 94 °C for 30 s. annealing and extension at 72 °C for 1 min. Followed by an additional extension at 72 °C for 10 min at the end of the 35 cycles. All PCRs included nontemplate controls. Different combinations of Illumina adapters N701-N712 and [N/S/E]501-[N/S/E]508 and [N/S/E]517) were used. introducing index and variable sequences. sequencing primer binding sites and regions complementary to the flow cell Oligos which are critical for cluster generation. The combination of index and variable sequences in forward and reverse primers are unique to each set of primer pair and serve as identifiers for each study subject. As they are specific to a given sample library they enable multiple sequences to be sequenced together and are used for de-multiplexing during data analysis to assign individual sequence reads to the correct sample during final data analysis.

1.10 Table S10. Genotyping

5-HTTLPR genotype was determined using PCR (T100, Biorad, USA) and subsequent gel electrophoresis. The PCR conditions and reagents are shown in the table. The samples were then applied to a 2.5% agarose gel for 90 minutes at 120 voltage.

FKBP5 genotype of rs1360780 was evaluated using high resolution melt analysis (HRM; CFX 384 Well Real-Time PCR System, Biorad, USA). HRM is a quantitative analysis of the melt curves of product DNA fragments following PCR amplification. Thereby, the combination of qPCR instrumentation and saturating DNA-binding dyes allows for the identification of small variations in nucleid acid sequences by the controlled melting of double-stranded PCR amplicons.

**Gene**		**5-HTTLPR (*SLC6A4*)**	**rs1360780 *(FKBP5)***
**Primer**	forward	tcctccgctttggcgcctcttcc	AATATCTCTTGTGCCAGCAGTAG
	reverse	tgggggttgcaggggagatcctg	CAGAAGAGATCCAGGCACAGA
**Length [bp]**		512/469	90
**PCR Reagents**		4µl 5X Green GoTaq Reaction Buffer (Promega, USA)<br>1.2 µl $MgCl_2$(25mM)<br>0.4 µl Primer (10µM)<br>0.4 µl dNTPs (10µM)<br>0.15 µl Taq (5units/µl)<br>11.35 µl H2O<br>2.5 DNA (20ng/µl)	5µl HRM-Mix (2x), Precision Melt Supermix (Biorad, USA)<br>0.2µl Primer (10µM)<br>3.8µl H2O<br>1 µl DNA (20ng/µl)
**PCR Conditions**		94°C 3:00<br>94°C 0:30<br>65.5°C 1:30<br>72°C 1:00<br>Go to step 2 39x<br>72°C 10:00<br>12°C ∞	95°C 5:00<br>95°C 0:05<br>60°C 10<br>Go to step 2 40x<br>65°-95°C in 0.2°C steps

1.11 Table S11. Permutation tests

Gene	Skew	Kurtosis	p-value		
			Time	Time × responder	Correlation with ΔPCL
FKBP5	0.25	-0.58	.533	.771	.776
SLC6A4	-0.96	0.89	.256	.929	.872
OXTR	-0.89	0.70	.142	.462	.386
NR3C1	0.17	0.09	.286	.143	.232
ADORA1	0.87	0.59	**.035**	.463	.154
DMR-1	-0.23	-0.25	.860	.628	.440
RPS6KA2	-0.18	1.39	.920	.290	.344
TSPAN9	-0.98	0.91	**.050**	.405	.222

Note. Skew and kurtosis are reported here for the difference scores between T2 and T1. Permutation tests were conducted using the ezPerm function of the ez package (v4.4.0) for the ANOVA tests and with a custom function for the correlations, randomly resampling one of the two variables without replacement. In either case, 1000 permutations were performed. P-values correspond to those reported in Table 1 and Table 2. P-values < .05 shown in bold (uncorrected).

2. Supplementary Figures

2.1 Figure S1. Chromosomal location of analyzed regions of the PTSD cohort

Chromosomal positions of analyzed genomic regions are displayed using graphical output from the UCSC genome browser. All genes are shown in 5´→3´ orientation from left to right. The analyzed DNA regions are shown as red bars. CpG islands with the number of CpGs are shown in green bars.

A) <u>Candidate Genes</u>

2.1.1 NR3C1

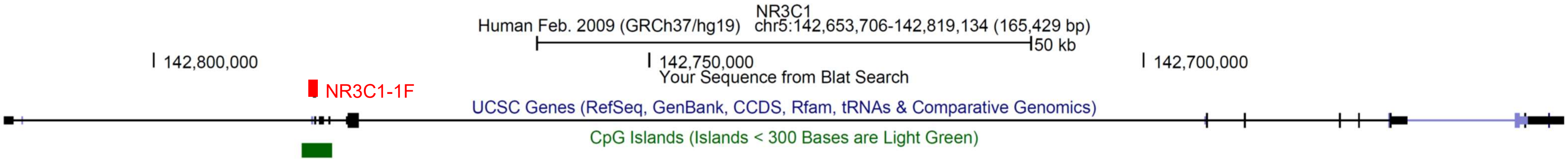

NR3C1: DNA methylation of 42 CpGs in the promoter region of exon 1F of *NR3C1* (Glucocorticoid Receptor) was analyzed (chr5:142.783.541-142.783.911).

2.1.2 SLC6A4

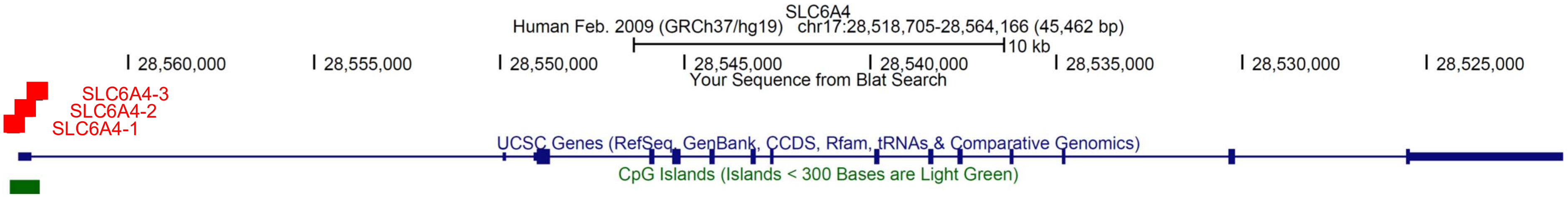

SLC6A4: DNA methylation of the entire CpG island located in the promoter region of the *SLC6A4* (Serotonin Transporter) was analyzed. The CpG island was divided into 3 fragments for sequencing (SLC6A4-1:chr17:28.562.939-28.563.283; SLC6A4-2: chr17:28.562.574-28.562.952; SLC6A4-3: chr17:28.562.328-28.562.682). CpG sites which are affected by SNPs were not included in the statistical analysis (rs25533; rs35206195; and rs56384968).

2.1.3 *OXTR*

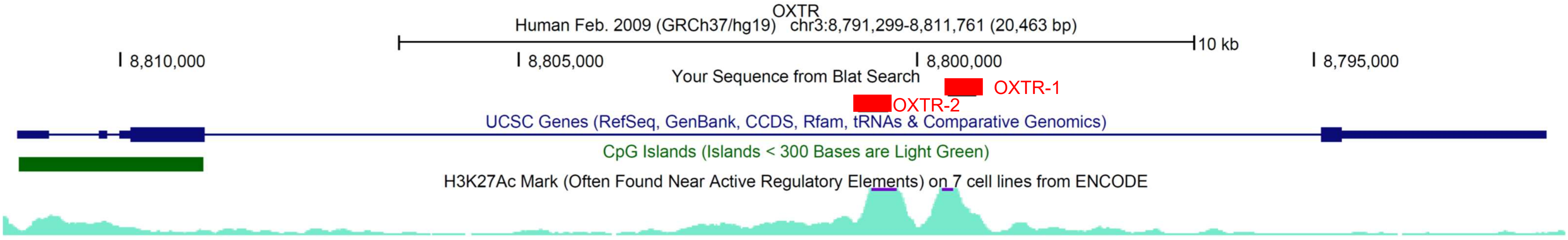

OXTR: 16 CpGs in a putative *OXTR* (Oxytocin Receptor) enhancer region (intron 3) were analyzed (OXTR-1: chr3:8.799.262-8.799.615; OXTR-2: chr3:8.800.371-8.800.739). This region is characterized by histone acetylation (H3K27Ac: turquoise peaks). CpG sites which are affected by SNPs were not analyzed in the statistical analysis (rs2268491 and rs7636061).

2.1.4 *FKBP5*

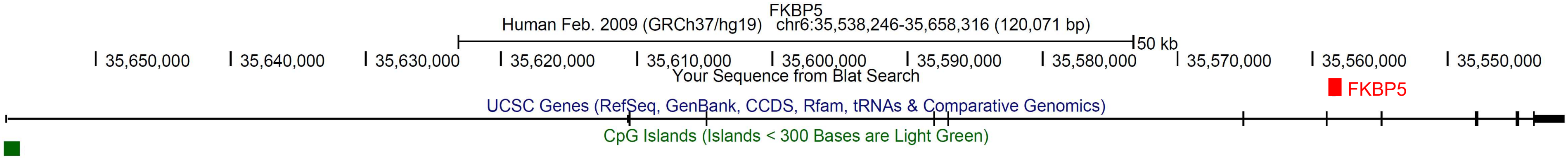

FKBP5: DNA methylation analysis was performed on 5 CpGs in intron 7 of *FKBP5* (FKBP Prolyl Isomerase 5) containing a glucocorticoid response element (GRE) (chr6:35.558.322-35.558.593 272).

B) New Targets from WGBS

2.1.5 ADORA1

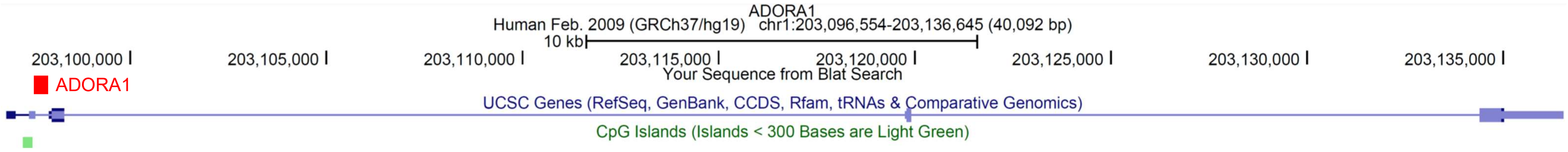

ADORA1: DNA methylation of 7 CpGs in *ADORA1* (Adenosine A1 Receptor) was analyzed (chr1:203.097.582-203.097.833).

2.1.6 TSPAN9

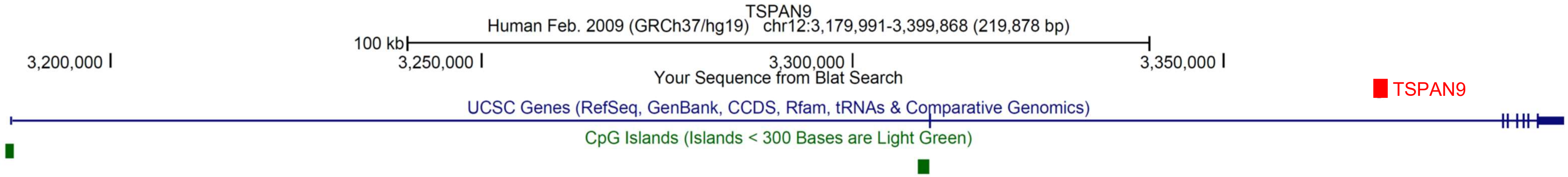

TSPAN9: DNA methylation of 6 CpGs in *TSPAN9* (Tetraspanin 9) were analyzed (chr12:3.370.787-3.371.092).

2.1.7 RPS6KA2

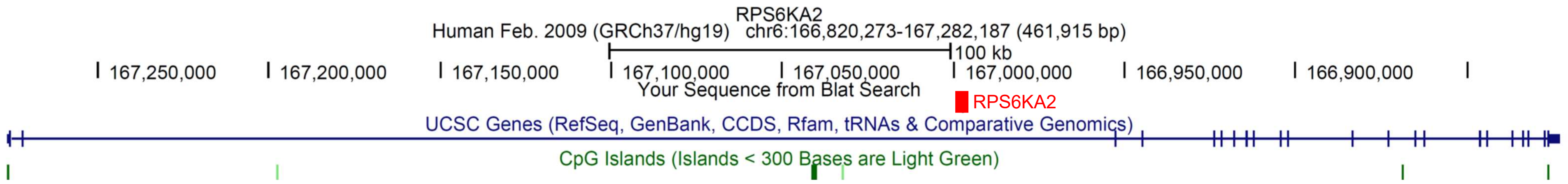

RPS6KA2: DNA Methylation of 8 CpGs in *RPS6KA2* (Ribosomal Protein S6 Kinase A2) was analyzed (chr6:166.996.725-166.997.045).

2.1.8 DMR-1

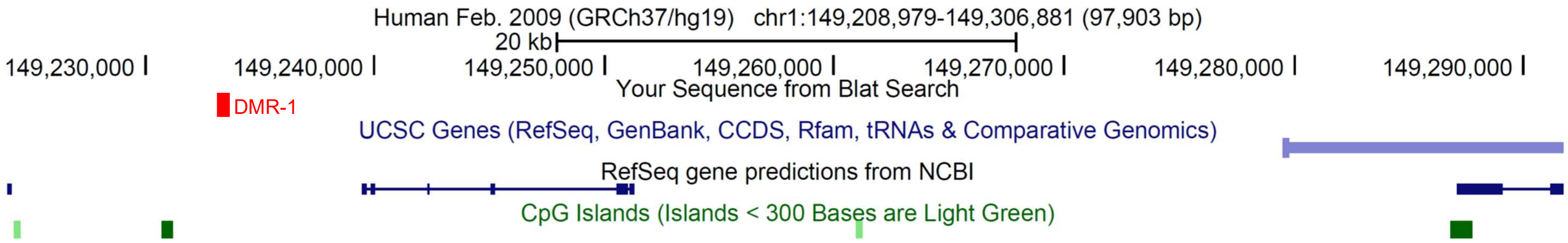

DMR-1: DNA methylation of 14 CpGs was analyzed according to the WGBS results. The differentially methylated region (DMR) is about 9 kbp downstream of *RNVU1-18* (RNA. Variant U1 Small Nuclear 18). 6 kbp upstream of *LOC105369140* (NBPF Member 6 Pseudogene) and ~50 kbp upstream of Long Intergenic Non-Protein Coding RNAs (*LOC388692* and *LOC644634*). *RNVU1-18* is affiliated with the snRNA class. *LOC105369140* is a pseudogene. Location of DMR-1: chr1:149.233.322-149.233.624. CpG sites which are influenced by SNPs were not analyzed (rs2319160 and rs2319163).

2.2 Figure S2. Correlations between symptom change and DNA methylation change in the PTSD cohort

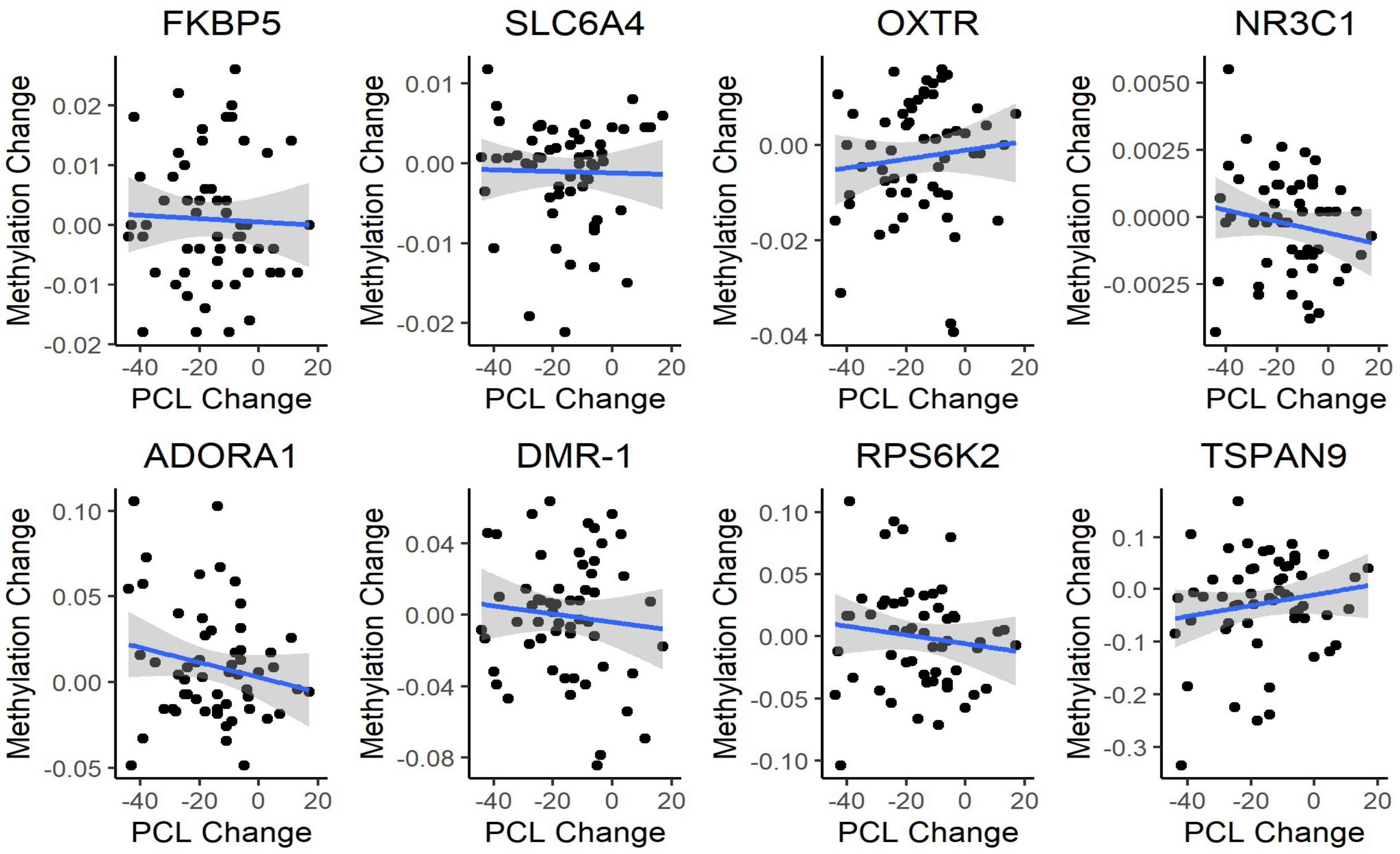

2.3 Figure S3. Principal component analysis

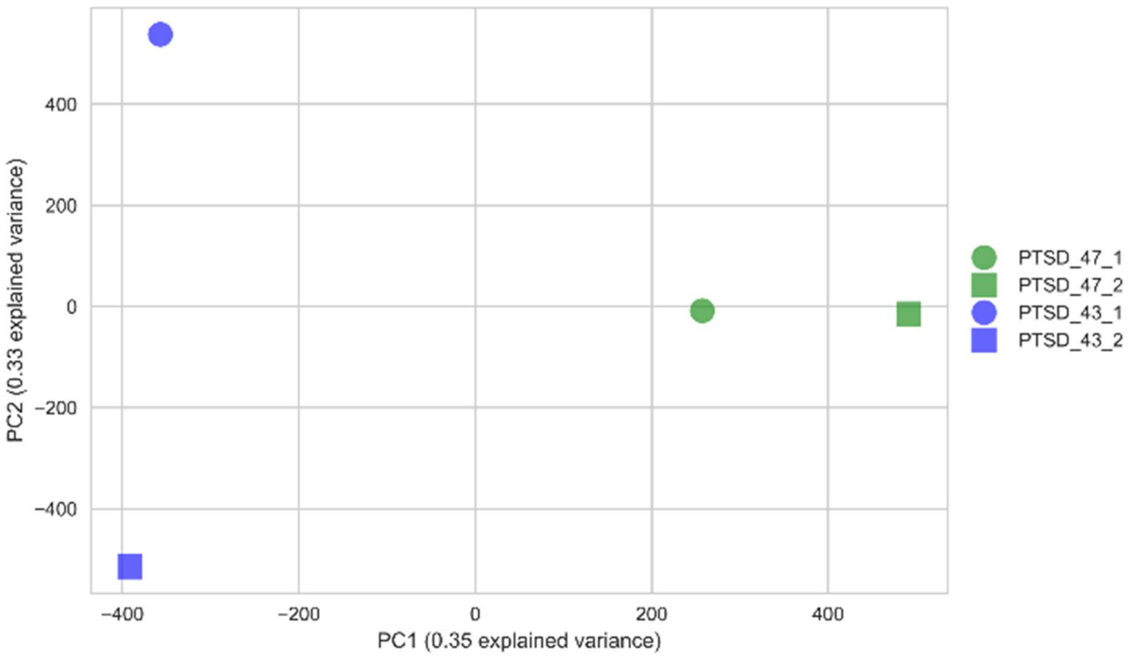

Figure S3. Principal component analysis. (PCA): PCA of the four PTSD datasets (2.8 million CpGs). Only CpG loci with minimum coverage of 10 reads in all samples and minimum mapping quality of 30 are considered.

3. Supplementary Methods

3.1 Methods S1. Statistical analysis

In the PTSD cohort, repeated measures analyses of variance (ANOVA) were performed to assess changes in DNA methylation from pre- to post intervention in selected candidate genes as well as WGBS nominated novel targets. Therapy response was included as an additional between-subject factor to check for therapy outcome-dependent changes in DNA methylation. Moreover, Pearson correlations were computed between percent DNA methylation change and PCL-5 symptom change to present a continuous measure of therapy response. P-values derived from both approaches, categorical and continuous, were supplemented with Bayes factors to quantify the evidence for the null hypothesis using the BayesFactor package (v0.9.12-4.2) in R (3.6.1) and non-informative default priors. The Bayes factor divides the likelihood of the data given a model without the effect of interest by the likelihood of the data given a model including the effect of interest. If the data is more likely under the null hypothesis, the Bayes factor becomes larger than 1. Classically, Bayes factors above 3 are interpreted to represent substantial evidence in factor of the null hypothesis, although this convention should be viewed as a rough guideline instead of a definitive cutoff.

Complementary to the Bayesian approach, we conducted equivalence tests to assess whether effect sizes in DNA methylation change are significantly smaller than the smallest biologically meaningful effect size. It has been argued that DNA methylation below 5% should be interpreted with extreme caution. We used the two one-tailed t-test procedure to check whether empirical effect sizes for methylation change are smaller than 5% or even a more conservative 1%. These tests were conducted for both the whole cohort and the subgroup of therapy-responders. This approach is useful here, as the Bayes factor approach only evaluates the evidence against the existence of a standardized effect size (e.g. Cohens d), which represent a ratio between signal and noise. If both signal and noise are small, i.e. when methylation is relatively stable over time, equivalence tests against an unstandardized theoretically meaningful effect size (i.e. percent methylation change) can be more powerful.
